# Integration of Ontogeny-Based Changes for Predicting the Exposure of Diphenhydramine in the Pediatric Population: A PBPK Modeling Approach

**DOI:** 10.3390/pharmaceutics16121553

**Published:** 2024-12-04

**Authors:** Ammara Zamir, Muhammad Fawad Rasool, Faleh Alqahtani, Hussain Alqhtani, Tanveer Ahmad

**Affiliations:** 1 Department of Pharmacy Practice, Faculty of Pharmacy, Bahauddin Zakariya University, Multan 60800, Pakistan; ammarazamir20@gmail.com; 2 Department of Pharmacology and Toxicology, College of Pharmacy, King Saud University, Riyadh 11451, Saudi Arabia; 3 Department of Clinical Pharmacy, College of Pharmacy, Najran University, Najran 61441, Saudi Arabia; hmhalqhtani@nu.edu.sa; 4 Institute for Advanced Biosciences (IAB), CNRS UMR5309, INSERM U1209, Grenoble Alpes University, 38700 La Tronche, France

**Keywords:** pharmacokinetics, PBPK modeling, pediatrics, diphenhydramine, ontogeny, cytochromeP450

## Abstract

Background: Diphenhydramine is an anti-tussive used periodically to treat seasonal colds, contact dermatitis, and anaphylactic reactions. This study aimed to develop a physiologically based pharmacokinetic (PBPK) model of diphenhydramine in predicting its systemic exposure among healthy pediatrics (children and adolescents) by leveraging data files from adults (young and elderly). Methods: The data profiles comprising serum/plasma concentration over time and parameters related to diphenhydramine were scrutinized via exhaustive literature analysis and consolidated in the PK-Sim software version 11.1. This modeling methodology commences with developing an adult model and then translating it to the pediatrics which compares the predicted concentration–time datasets with the reported values. Results: The accuracy of model anticipations was then assessed for each pharmacokinetics (PK) variable, i.e., the area under the curve from 0 to infinity (AUC_0-∞_), maximal serum/plasma concentration (C_max_), and clearance of the diphenhydramine in plasma (CL) by employing the predicted/observed ratios (R_pre/obs_), and average fold error (AFE), which fell within the pre-defined benchmark of 2-fold. The predicted and observed C_max_ values for pediatrics were 3-fold greater in comparison to the young adults following a 25 mg dose depicting a need to monitor dosage schedules among children closely. Conclusions: These model-based anticipations confirmed the authenticity of the developed pediatric model and enhanced the comprehension of developmental variations on PK of diphenhydramine. This may assist healthcare professionals in ensuring the significance of lifespan applicability in personalized dose regimens, promoting therapeutic efficacy and minimizing side effects in chronic conditions among children.

## 1. Introduction

Since the last decade, physiologically based pharmacokinetic (PBPK) modeling has illustrated notable progress in various drug emergence and regulation phases by elucidating their liberation, absorption, and disposition in the human body across the whole range of populations, i.e., from pediatrics to the elderly [1,2,3,4]. The increased publications regarding modeling and simulation techniques have paved the way for the consideration of the recommendations in research and development (R & D) of drugs [5]. The knowledge constraints among children regarding specific dosage schedules and formulations have increased the practice of off-label drug use leading to life-threatening problems [6]. Contrary to the previous semi-mechanistic and top-down modeling approaches among which weight and surface area of the body were used to anticipate the doses of different medications in children, PBPK models summarize the complicated mechanism and age-based drug behaviors in the human body towards a physiologically pragmatic compartmental framework [4]. Therefore, the evolution of new mathematical techniques supporting the PBPK approach for characterizing the dose-response relationships allows for the planning and execution of insightful pediatric trials [7].

The clinical research studies among the pediatric population are more problematic than those of young adults due to ethical and practical limitations [8]. The European Medicine Agency (EMA) and the United States of America (USA) have initiated regulations to promote the use of medications in children leading to greater concern and recognition in research [9,10]. This has made PBPK modeling come up with an intriguing role by narrowing the gap in the pharmacology of adults and children [5]. The combination of in silico data from the literature, physicochemical parameters of drugs, expression of enzymes, and population-based information provides quantitative anticipation of the pharmacokinetics (PK) in humans [11]. Furthermore, these models have the potential to integrate data on the alterations in development and growth concerning age, including blood flows, the composition of the individual, and variance in ontogeny patterns for disposition [4]. Several clinical research articles on PBPK models of various drugs in pediatrics were published in the last decades [12,13,14,15].

Diphenhydramine is a derivative of ethanolamine and belongs to the class of histamine-1 (H-1) receptor antagonists which is indicated in the treatment of seasonal rhinitis, insect bites, pruritis, allergic rashes, insomnia, vertigo, dystonias, and motion sickness [16,17]. It is a widely used over-the-counter (OTC) medication for inducing sleep in patients suffering from the common cold [18]. It works by binding inversely with the H-1 receptor thus antagonizing the action of histamine on various sites in the body leading to lessening of allergic reactions, inducing drowsiness, and reducing cough symptoms [17,18]. Diphenhydramine is available in tablet, capsule, and syrup formulations via the per-oral (PO) route and as a bolus via the intravenous (IV) route [18,19,20,21]. The Biopharmaceutical Classification System (BCS) has assigned class I to diphenhydramine [22] depicting its high solubility of 3.06 mg/mL at pH 7.00 [23]. Diphenhydramine undergoes metabolism by N-demethylation which is catalyzed by various cytochrome P450 (CYP450) enzymes such as CYP2D6, CYP1A2, CYP2C9, and CYP2C19 [24] and eliminated in the urine with renal clearance (CL_R_) of 0.01 L/h/kg [25].

The Food and Drug Administration (FDA) approved the use of diphenhydramine in pediatrics via IV [26] and the PO route [27] excluding the pre-term and neonate population. Different anatomical and physiological changes occur in children, such as weights of organs, the content of fat and water in the body, cardiac output, the composition of tissues, blood flow rates, plasma protein levels, and development processes including variations in functions of enzymes and renal elimination [12,28,29]. Due to these ontogeny-based alterations, developing a PBPK model for diphenhydramine in this special population (children) may provide the basis for anticipations of its PK and thus may aid in preventing adverse drug events beforehand.

This clinical research study was conducted because only one PBPK model had been built in the past among adults in which diphenhydramine and caffeine were utilized as case drugs [30]. Moreover, no PBPK model in the pediatric population has been presented in the literature up till now. In 2018, a study of diphenhydramine in children aged 2–18 years was published focusing on its PK by employing dosage regimens based on age and weight [18]. The goal of this research study was first to develop and evaluate the PBPK model of diphenhydramine in healthy adults and elderly populations and then scale it among pediatric subjects. Second, the genotypic effect on exposure to diphenhydramine among children (variant age groups) was observed at the reported PO dose. Third, the variant systemic exposure of diphenhydramine in different stages of chronic kidney disease (CKD) and liver cirrhosis (LC) was demonstrated among adults to correlate them with children. This may help clinicians to utilize evidence-based perceptions to optimize the age-dependent dosage schedules, provide threshold safety, and thus minimize the risk of overdose and drug–drug interactions.

## 2. Materials and Methods

### 2.1. Scavenging of Reported PK Data

The medical subject heading (MESH) terms such as “pharmacokinetics”, “diphenhydramine”, and “humans” were employed in Google Scholar, and PubMed online databases to extract the relevant studies comprising serum/plasma systemic concentration–time graphs on diphenhydramine after PO and IV routes of administration in young, elderly, and pediatric populations. The quality of the included datasets was based on ethical approval, the method of analysis, and the presence of details for the study protocol. The datasets were excluded if they were comprised of pre-term and neonate populations. The clinical studies demonstrating population cohort, age range, respective body weights, sample size, females proportion, applied dose, and application route were added to the model whose characteristics are represented in Table 1. Among 12 profiles (1 IV bolus and 11 PO) from 4 datasets, 5 profiles were reported in young adults (men and women), 3 were in elderly adults (men and women), and 4 were in children and adolescents. The PBPK model was developed and verified by employing one-third (1 IV, 4 PO) and two-thirds (4 PO) datasets, respectively. In contrast, all data profiles were utilized in the final evaluation of model performance.

### 2.2. Software for Development of the PBPK Model

PBPK modeling was executed by software such as SimCyp^®^ version 23, PK-Sim^®^ version 11.1, GastroPlus^®^ version 9.9, etc. in the previously reported research articles but PK-Sim^®^ version 11.1 (Bayer Technology Services, GmbH Systems Biology Software Suite Wuppertal, Germany) [31] was selected in this PBPK model, due to its user-friendly interface, easy access, advanced algorithms, and updated version with the integration of in-built CKD population and ontogeny changes. The absorption, distribution, metabolism, and elimination (ADME) of diphenhydramine were anticipated in adult, elderly, and pediatric populations using PK-Sim^®^. GetData Graph Digitizer version 2.26 software [32] was utilized to scan all previously reported graphs to convert their digital format into arithmetic values.

### 2.3. Construction of Building Blocks

The Open System Pharmacology Suite (OSP) has assembled the interface of PK-Sim^®^ software that embraces expression profiles of metabolizing enzymes and transporters, individuals, respective populations, compounds of relevant drugs, and formulation in case of the PO route, administration protocols for single or multiple doses, food events, and reported observed data. The values for percentage coefficient of variation linked with CYP1A2, CYP2C9, CYP2C19, and CYP2D6 were maintained in adults to account for inter-individual variability which predicts the disposition of diphenhydramine in simulations of children and adolescents [33].The details of data files in adults (young and elderly), and pediatric populations, physicochemical properties (lipophilicity (Log P), solubility, etc.), and ADME of diphenhydramine were extracted from previously published clinical trial studies whose particulars are detailed in Table 2.

### 2.4. Framework for Building the PBPK Model

**Figure 1:**
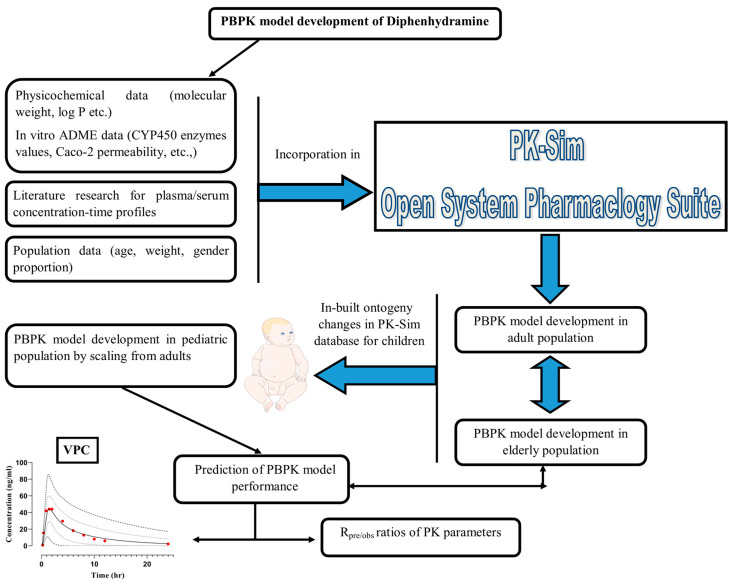
Schematic framework for PBPK model development of DPH in adults and pediatrics PBPK: Physiologically based pharmacokinetic modeling, DPH: Diphenhydramine, ADME: Absorption, distribution, metabolism, and elimination, Log P: Lipophilicity, CYP450: Cytochrome P450 enzymes, VPC: Visual predictive checks, R_pre/obs_: Predicted/observed ratios. The image of the child was incorporated from “Servier Medical Art” https://smart.servier.com/ (accessed on 12 November 2024).

A systematic workflow was used to develop the PBPK model on diphenhydramine encompassing a detailed literature screening for PK data profiles, and drug-specific ADME parameters. The combination of both of these with the population data was then embodied into the PK-Sim^®^ OSP suite to build up the model in healthy young, elderly, and pediatric populations by following the protocols of recently published research papers [36,37]. First, the model was built in the adult (young and elderly) population after the IV route followed by PO to understand the baseline parameters of diphenhydramine. After this, the model was scaled to the children by utilizing in-built physiological, biochemical, anatomical, and ontogeny-related changes in PK-Sim^®^. The graphical illustration of this adult and pediatric PBPK model development process is represented in Figure 1.

### 2.5. Model Structure

**Figure 2:**
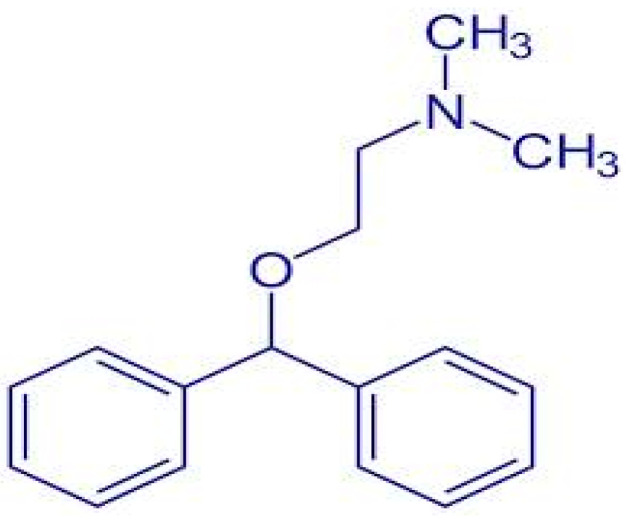
Chemical structure of Diphenhydramine.

Diphenhydramine is a pure white powder with a chemical formula of C_17_H_21_NO and a log P of 3.27 [16,25]. The ionization constant (pKa) of 8.98 depicts its weak basic nature which is due to its aliphatic tertiary nitrogen atom (Figure 2). The fraction unbound of 18% was employed in the model. Moreover, despite the in-built absorption model, the Caco-2 cell permeability of 5.43 × 10^−4^ cm/s was integrated [30] whereas PK-Sim software computed the specific organ permeability as 0.19 cm/min. The Rodger and Rowland and PK-Sim standard methods were used to evaluate the cellular permeability and partition coefficient. In the case of the PO route, the formulation method of Lint80 was used in which adjustment was made in the dissolution time factor (45 min) to enhance the precision of adult and pediatric PBPK models. The other variables are listed in Table 2.

### 2.6. Scaling of Adult PBPK Model to Pediatrics

The adult diphenhydramine PBPK model was then scaled to the pediatric population for examining the alterations in ADME of children with ages ranging from 2 to 11 years. The age-related changes in weight, height, and body mass index were incorporated in the model whereas the other anatomical, physiological (blood flows, volumes, and sizes of various organs, cardiac output, hematocrit, levels of albumin) parameters, aqueous and lipid content in the body, changes in concentrations of CYP1A2, CYP2D6, CYP2C9, and CYP2C19 enzymes, and alterations in glomerular filtration were utilized from the in-built PK-Sim^®^ OSP suite database. The World Health Organization (WHO) has categorized the pediatric ages into different stages such as neonates (from birth to 28 days), infants (28 days to 1 year), young children (1 to 4 years), toddlers (2 to 3 years, older children (5 to 12 years), and adolescents (12 to 18 years) [38]. Box plots were made to present the exposure of diphenhydramine in all ages from 2 to 18 years initially in comparison to the adults. Afterward, 0 expression was integrated in all enzymes (CYP1A2, CYP2D6, CYP2C9, and CYP2C19) individually and then the differences in systemic exposure among adults and the pediatric age range were shown by making box plots. Finally, a graphical display of box plots was utilized to represent the exposure variance in adults among all stages of CKD and LC; thus, based on this, assumptions in alterations among the pediatric subjects can be made.

### 2.7. Verification of Model

A virtual population cohort (N = 1000) was built in PK-Sim OSP software for adults (young and elderly), children, and adolescents by employing all information from related datasets containing systemic concentration–time graphs (mean and standard deviation), demographic variables, and dosage protocols. The age groups ranging from 2.76–10.6 yr (in children), 12.75–16.45 yr (in adolescents), 21.1–31.8 yr (in young adults), and 62.5–73.7 yr (in elderly adults) were included in this model. The proportion of females was inputted as mentioned in the literature (Table 1). In the absence of an explanation for gender percentage, 50% of the total sample size was set as default for females as mentioned in a previous study [39]. The PBPK analysis of diphenhydramine was then assessed by visual predictive checks (VPC) where published data were superimposed on the anticipated data along with the arithmetic mean, 5th to 95th centile values, and range of concentration (i.e., minimum and maximum). The PK-Solver (Microsoft Excel Adds-in version 2013) program [40] was then used to execute the non-compartmental analysis (NCA) for scavenging PK parameters such as maximal serum/plasma concentration (C_max_), area under the curve from time 0 to infinity (AUC_0-∞_), and clearance of diphenhydramine in serum/plasma (CL) for both simulated and reported data. The R_pre/obs_ along with 95% CI, and average fold error (AFE) for all PK variables (C_max_, AUC_0-∞_, and CL) was computed to enhance the model reliability by applying Equations (1) and (2) displayed below:

(1) $$R=\;\frac{Predicted\;value\;of\;PK\;parameter}{Observed\;value\;of\;PK\;parameter}$$

(2) $$AFE=10^{\frac{\sum \log\left(fold\;error\right)}{N}}$$

## 3. Results

### 3.1. PBPK Model Evaluation in Adults (Young and Elderly)

**Figure 3:**
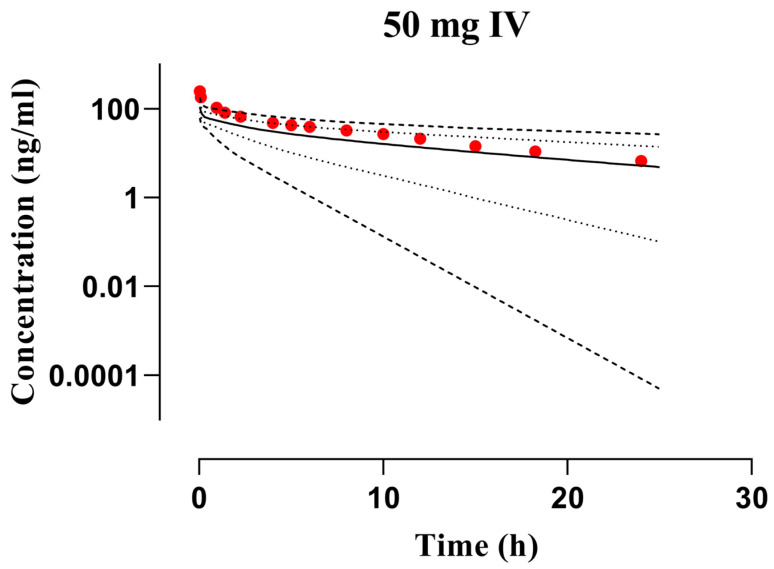
Observed and simulated plasma concentration data files of diphenhydramine with respect to time in young adults after the application of intravenous bolus dose of 50 mg [19]. The reported and anticipated data are represented by red circles and solid lines, whereas the range, i.e., minimum and maximum, and 5th to 95th centiles are depicted by dashed and dotted lines, respectively. IV: Intravenous.

**Figure 4:**
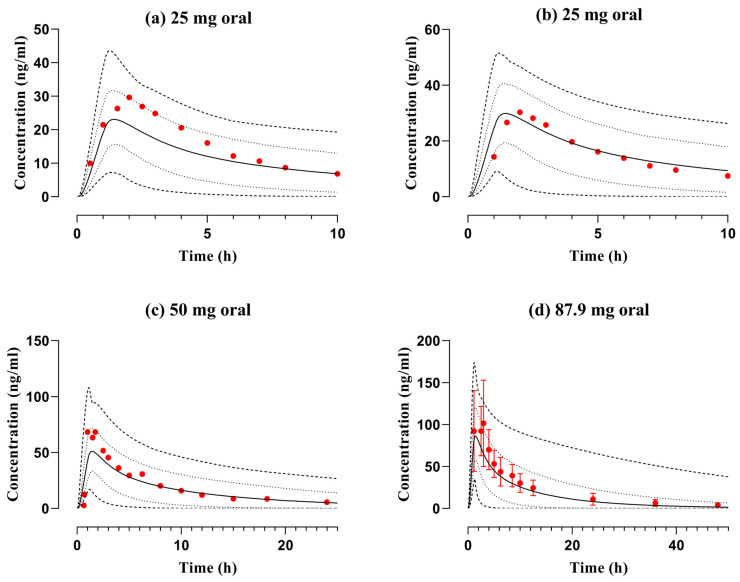
Observed and simulated plasma concentration data files of diphenhydramine with respect to time after application of PO dose of (**a**,**b**) 25 mg [21] in young men and women, (**c**) 50 mg [19], and (**d**) 87.9 mg [20] among young adults. The reported and anticipated datasets are represented by red circles and solid lines, whereas the range, i.e., minimum and maximum, and 5th to 95th centiles are depicted by dashed and dotted lines, respectively. Standard deviation is portrayed by error bars in the reported data of graph (**d**).

**Figure 5:**
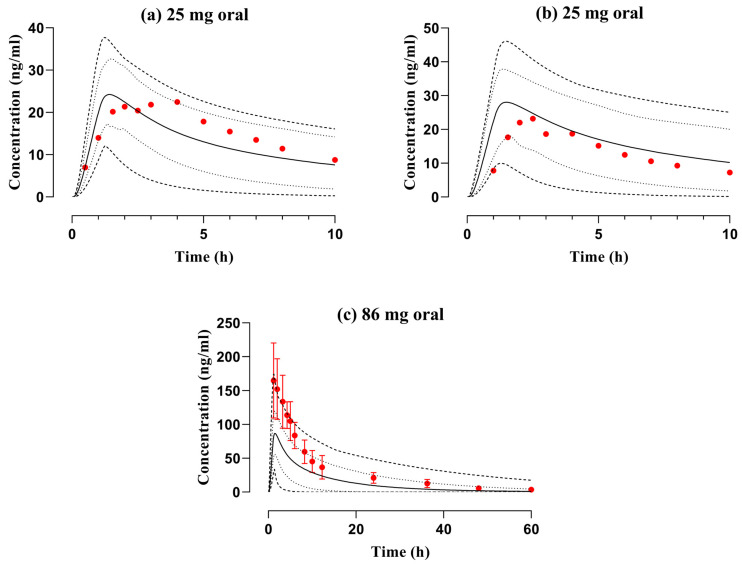
Observed and simulated plasma concentration data files of diphenhydramine with respect to time after application of PO dose of (**a**,**b**) 25 mg [21] in elderly men and women, and (**c**) 86 mg [20] among elderly adults. The reported and anticipated datasets are represented by red circles and solid lines, whereas the range, i.e., minimum and maximum, and 5th to 95th centiles are depicted by dashed and dotted lines, respectively. Standard deviation is portrayed by error bars in the reported data of graph (**c**).

The PBPK model-anticipated diphenhydramine serum/plasma concentration–time data files among young adults after administration of 50 mg IV bolus dose (Figure 3) and 25–87.9 mg PO dose in both young and elderly adults (Figure 4 and Figure 5) were compared with reported mean concentration data. The observed and predicted findings were closely identical regarding arithmetic mean and 5th to 95th centiles. Furthermore, mean R_pre/obs_ ratios and average fold error (AFE) were computed to verify the accuracy of the developed PBPK model of diphenhydramine. The AFE for C_max_ was 0.83 in young adults and 0.92 among elderly adults depicting to fall within the pre-established acceptance criterion, i.e., 0.5–2-fold error range. The pediatric PBPK model was effectively verified with 2-fold error in R_pre/obs_ and a value of nearly 1 in the case of AFE which is regarded to be a logical prediction by the World Health Organization (WHO) [41] and also from previously published research articles [14,42,43]; so, values outside this range would not be considered reliable. The values for residual PK variables can be discerned in Table 3 and Table 4.

### 3.2. PBPK Model Evaluation in Pediatrics and Adolescents

**Figure 6:**
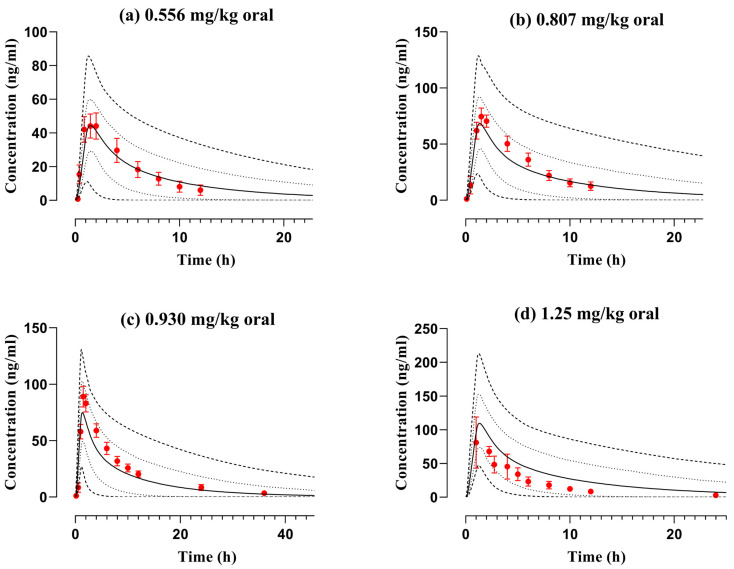
Observed and simulated plasma concentration data files of diphenhydramine with respect to time after application of PO dose of (**a**) 0.556 mg/kg [18], (**b**) 0.807 mg/kg [18], (**c**) 0.930 mg/kg [18], in children and adolescents, and (**d**) 1.25 mg/kg [20] in children. The reported and anticipated datasets are represented by red circles and solid lines, whereas the range, i.e., minimum and maximum, and 5th to 95th centiles are depicted by dashed and dotted lines, respectively. Standard deviation is portrayed by error bars in the reported data of graphs (**a**–**d**).

The PBPK model was developed among children with age categories of 2–5 years, 6–11 years, and adolescents with ages ranging from 12–18 years which depicted that the published observed data profile was in line with simulated data after the application of PO doses of 0.556 mg/kg, 0.807 mg/kg, 1.25 mg/kg (in children), and 0.930 mg/kg (in adolescents). The visual illustration of the predicted and reported data along with the arithmetic mean, the range of minimum and maximum, and the 5th to 95th centiles are represented in Figure 6. In addition, the mean R_pre/obs_ ratios and AFE values were calculated using pre-defined equations and presented in tabular format to enhance the reliability of the diphenhydramine model in pediatrics. The AFE value for AUC_0-∞_ among children and adolescents after PO application was 1.11, whereas the other residual PK parameters were also within the ideal 2-fold error range (Table 3 and Table 4).

### 3.3. Assessment of Dose-Exposure Relationship Among Adults and Pediatrics in Various Scenarios

**Figure 7:**
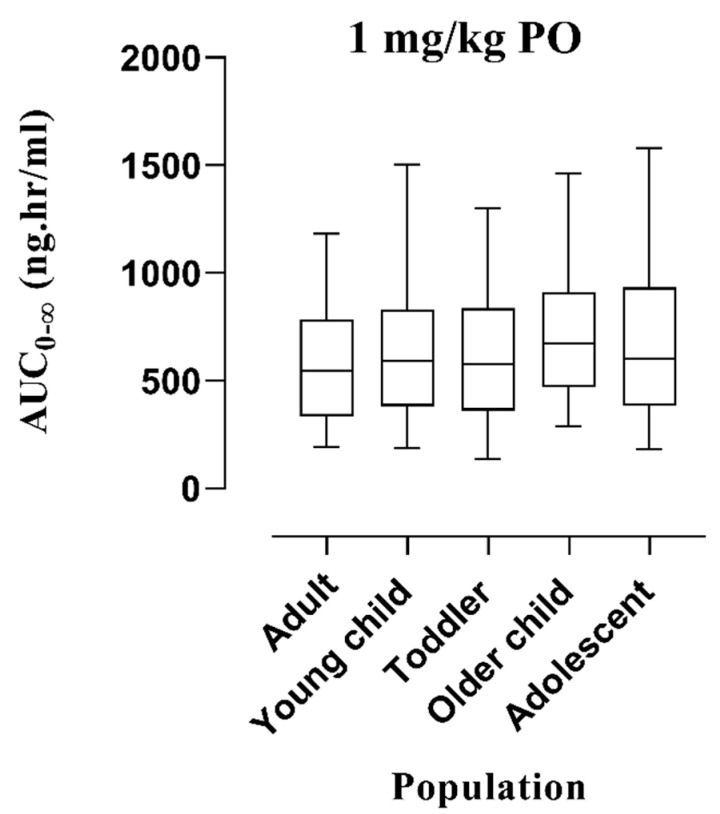
Visual depiction of anticipated AUC_0-∞_ with 5th to 95th centile by employing box whisker plots after administering a PO dose of 1 mg/kg in the adult population, and pediatrics of different age ranges as per guidelines of the World Health Organization. PO: Per-oral, AUC_0-∞_: Area under the concentration–time curve from 0-∞.

**Figure 8:**
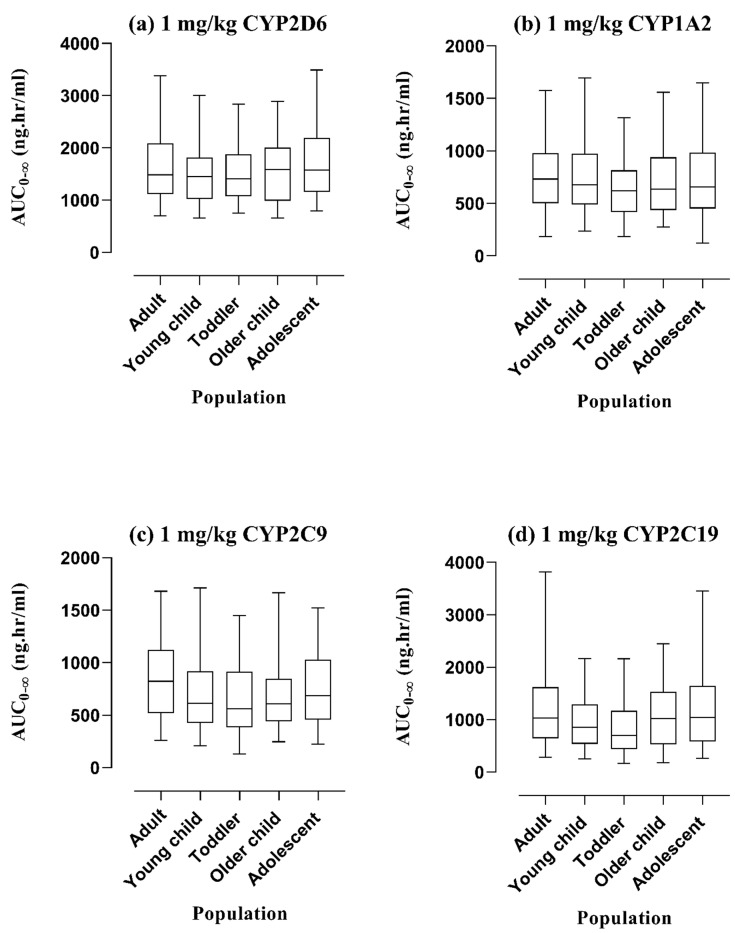
Visual depiction for comparison of anticipated AUC_0-∞_ with 5th to 95th centile to exhibit the impact of CYP2D6, CYP1A2, CYP2C9, and CYP2C19 by employing box whisker plots after administering PO dose of 1 mg/kg in the adult population, and pediatrics of different age ranges as per guidelines of World Health Organization. The expression of the relevant enzyme is set at zero in (**a**–**d**). AUC_0-∞_: Area under the concentration–time curve from 0-∞, CYP450: Cytochrome P450 enzymes.

**Figure 9:**
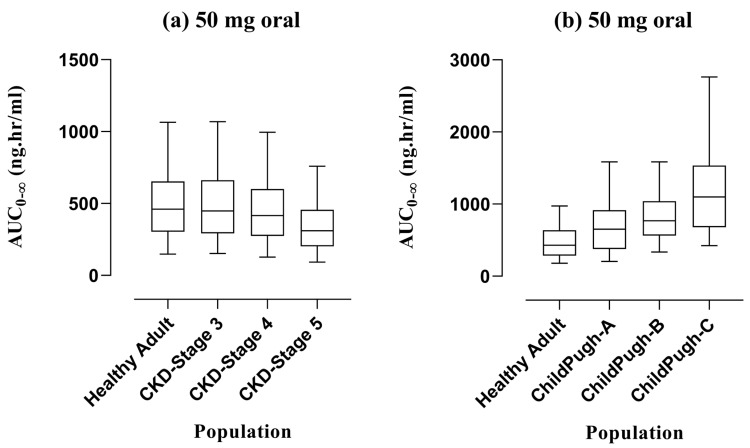
Visual depiction of anticipated AUC_0-∞_ with 5th to 95th centile by employing box whisker plots after administering a PO dose of 50 mg in a healthy adult population, and their comparison with various stages of chronic kidney disease and liver cirrhosis. AUC_0-∞_: Area under the concentration–time curve from 0-∞.

A 1 mg/kg PO dose was taken as a reference from the literature [27] for diphenhydramine among pediatrics and simulations were performed to exhibit the differences in AUC_0-∞_ among adults, young children (1–4 years), toddlers (2–3 years), older children (5–11 years), and adolescents (12–18 years) by graphical illustration of box whisker plots (Figure 7). Moreover, the effects of enzymes, i.e., CYP2D6, CYP1A2, CYP2C9, and CYP2C19 on the exposure of diphenhydramine among adults and children were elaborated by integrating zero expression in them one by one. The comparison of variations in the AUC_0-∞_ for all CYP enzymes was presented by box whisker plots with the 5th to 95th centile in Figure 8. Lastly, box whisker plots were created after a single administration dose of 50 mg via the PO route to compare the systemic exposure of healthy adults in the case of renal impairment, i.e., CKD stage 3, 4, and 5, and liver impairment, i.e., Child–Pugh (CP) A, B, and C as depicted in Figure 9.

## 4. Discussion

In this research study, the PBPK model for diphenhydramine among healthy adults (young and elderly), and pediatrics (children and adolescents) following IV and PO application modes was documented. This established framework was utilized to extrapolate the PBPK model from adults to pediatrics to determine the exposure to diphenhydramine across a variable age range. Currently, as per an extensive literature search, this is the first pediatric PBPK model for diphenhydramine to be presented. Initially, this model was developed among young and elderly subjects to define the comparison among all PK variables. This PBPK model was further expanded to pediatrics and adolescents by employing in-built physiological, and ontogeny-related alterations in the PK-Sim OSP database.

The disposition of diphenhydramine was elaborated by constructing the PBPK model in healthy young adults and the C_max_ was 51.0 ng/mL in PBPK model projections which was comparable to the reported dataset, i.e., 68.42 ng/mL after 50 mg PO dose. Furthermore, a study presented datasets separately for men and women in young and elderly adults at a 25 mg dose. The findings showed that the values of PK parameters (AUC_0-∞_, C_max_, and CL) of both observed and simulated data were analogous (Table 3). The AFEs for AUC_0-∞_ and C_max_ were 0.97 and 0.92 after PO application in young and elderly adults which showed that the developed PBPK model favorably comprehended the disposition of diphenhydramine.

Due to the advent of a recent publication discussing the PK of diphenhydramine in children and adolescents, this investigation encompassed the scaling of the adult PBPK model to the pediatric population at various doses. Following administration of 0.556 mg/kg PO dose the CL of mean anticipated and observed data profiles in age ranging from 2–5 years was 1.73 L/h vs. 1.79 L/h which seemed comparable. Moreover, in adolescents (age 12–18 years) the predicted value of C_max_ was 74.16 ng/mL, equivalent to the published one, i.e., 89.1 ng/mL. Furthermore, the AFE of CL is 0.97 among children and adolescents which further confirmed the good agreement in predicting ADME of diphenhydramine among the pediatric population. The observed and simulated values of CL showed a 34% decrease in adolescents as compared to pediatrics (Table 3) because the activity of enzymes (CYP1A2, CYP2C9, CYP2C19, CYP2D6) increases progressively with age [44].

Box whisker plots were created at a dose of 1 mg/kg PO among adults and pediatrics of different categories, i.e., young children, toddlers, older children, and adolescents which showed an increase in the AUC_0-∞_ in the later populations in comparison with adults (Figure 7) suggesting the need of dose monitoring in them. Moreover, diphenhydramine is metabolized via CYP2D6, CYP1A2, CYP2C9, and CYP2C19 enzymes illustrating its major impact on the exposure among the pediatric population. The expressions of enzymes were set to zero and the box whisker plots depicted that systemic exposures were almost comparable in adults and pediatrics (Figure 8), which may recommend that the cumulative effect of all enzymes has a greater dose-exposure relationship as depicted in Figure 7.

Diphenhydramine is an extensively albumin-bound drug due to which changes may occur in its systemic exposure among individuals suffering from CKD and LC. The literature on diphenhydramine in CKD and LC is scarce so box plots were made among adults compared to all stages of CKD and LC. The graphical representation depicted no change in healthy adults and those presented with stage 3 and stage 4 of CKD, but in the case of stage 5, which is end-stage kidney disease (ESKD), the exposure was lower. This may be due to low serum albumin levels in dialysis patients because the malnourishment factor is involved here as mentioned in a previous study [34]. In contrast, the AUC_0-∞_ was sequentially increased from healthy adults to CP-A, CP-B, and CP-C stages in LC which may suggest the tailoring of dosage schedules in pediatrics based on the assumptions among adults. Moreover, the FDA also labeled the drug to make adjustments in hepatic impairment which is now confirmed via the visual depiction of graphs.

Although this PBPK model has accurately defined the diphenhydramine PK in healthy adults (young and elderly), children, and adolescents as per our pre-defined 2-fold criteria for AFE, these model-based anticipations have several limitations. First, for the PBPK model assessment, plasma/serum concentration over time data profiles was analyzed point by point to assimilate reported data but that may not match the real-time data thus impacting the quality of findings. Second, studies on diphenhydramine in the pediatric population are scarce which may reduce the generalizability of outcomes. The scaling of adult PBPK models to pediatrics has constraints as data on the CYP enzyme expression, changes in fluid volume of the gastrointestinal tract, transporter expression, and intestinal bile flows are scarce [45]; so, therapeutic regimens among children may differ from those of adults thus impacting the reliability of results. Lastly, less data are available on LC and ESKD, and therefore, exposure in adults is presented assuming similar changes among children. Further population-based studies are required to comprehend the differences in PK among the pediatric population.

## 5. Conclusions

The presented PBPK model auspiciously anticipated the ADME of diphenhydramine across varied ages and doses in healthy adults and pediatric populations. The changes relevant to physiology, anatomy, and ontogeny of various CYP450 enzymes among pediatrics were incorporated via an in-built PK-Sim database to enhance the robustness of the model. Moreover, the alterations in AUC_0-∞_ were discerned in comparison among healthy adults and pediatrics as well as in adults in the case of LC and CKD, suggesting the requirement of optimizing dosage regimens and preventing adverse drug events.

## Figures and Tables

**Table 1:** Overview of demographics and dosage protocol of diphenhydramine in fitting PBPK model among adults, elderly, and pediatric population.

Sr. No.	Population Cohort	Age Range (Years)	Applied Dose (mg)	Application Route	Participation of Females (%)	Weight (kg)	Reference Citation
1-	Young Adults	26–41	50	IV	40	N/S	[19]
				PO			
2-	Young Adults	21.1–41.9	87.9 ± 12.4	PO	N/S	60.4–80.2	[20]
	Elderly Adults	65.1–73.7	86.0 ± 7.3			59.6–82.4	
3-	Young Men	24.6–36.2	25	PO	N/A	71.7–76.1	[21]
	Young Women	27–31.8				56.7–74.1	
	Elderly Men	62.5–66.1				66.5–77.3	
	Elderly Women	68.9–71.3				65.6–72.8	
1-	Children	7.2–10.6	39.5 ± 8.4 (1.25) *	PO	N/S	24.8–38.4	[20]
2-	Children	2.76–4.84	10.2 (0.556) *	PO	38	13.7–20.9	[18]
		6.61–9.99	24.2 (0.807) *		56.25	23.14–36.66	
	Adolescent	12.75–16.45	50 (0.93) *		50	46.24–l64.36	

IV: Intravenous, PO: Per-oral, N/A: Not applied, N/S: Not specified. * The doses are in mg/kg.

**Table 2:** Values of input parameters for developing whole-body PBPK model of diphenhydramine.

Model Input Variables	Compiled Values	Study Citation
Physicochemical characteristics		
Molecular weight (g/mol)	255.36	[25]
Lipophilicity (Log P)	3.27	[16]
Solubility in water (mg/mL)	3.06 at pH 7.00	[23]
Plasma protein	Human serum albumin	[34]
Acid dissociation constant (pKa)	8.98	[23]
Absorption		
Caco-2 cell permeability (cm/s × 10^−4^)	5.43	[30]
Distribution		
Specific organ permeability (cm/min)	0.19	Calculated by PK-Sim
Cellular permeability model	Rodgers and Rowland	
Partition coefficient model	PK Sim standard	
Percent of drug unbound to protein (f_u_)	18%	[35]
Disposition (Metabolism and Elimination)		
CYP1A2 V_max_ (pmol/min/pmol recombinant enzyme)	14	[24]
CYP1A2 K_m_ (uM)	295	
CYP2C9 V_max_ (pmol/min/pmol recombinant enzyme)	4.43	
CYP2C9 K_m_ (uM)	134	
CYP2C19 V_max_ (pmol/min/pmol recombinant enzyme)	11	
CYP2C19 K_m_ (uM)	55.7	
CYP2D6 V_max_ (pmol/min/pmol recombinant enzyme)	2.38	
CYP2D6 K_m_ (uM)	1.12	
Renal clearance (CL_R_) (L/h/kg)	0.01	[25]

N/A: not applied, CYP: cytochrome P450, K_m_: concentration of substrate at which half of the reaction rate occurs, V_max_: rate of reaction at its maximum.

**Table 3:** R_pre/obs_ ratios for PK variables of diphenhydramine among adults (young and elderly) and pediatric PBPK model datasets.

Sr. No.	Doses	C_max_ (ng/mL)			AUC_0-inf_ (ng/mL·h)			CL (L/h)			Clinical Interpretation
		Pre	Obs	R Ratio	Pre	Obs	R Ratio	Pre	Obs	R Ratio	
Young adults after IV administration											
1-	50 mg	150.54	251.1	0.6	841.09	565.97	1.48	59.44	88.34	0.67	Require close monitoring for dose adjustment
Young adults after PO administration											
2-	25 mg (men)	22.95	29.65	0.78	191.45	190.25	1	130.58	131.4	0.99	Promote current dosing regimen
3-	25 mg (women)	29.75	30.28	0.98	256.4	212.39	1.2	97.5	117.7	0.82	
4-	50 mg	51	68.42	0.75	484.36	507.32	0.95	103.22	98.55	1.04	
5-	87.9 mg	83.44	101.62	0.82	828.82	1131.12	0.73	106.05	77.71	1.36	
Elderly adults after PO administration											
6-	25 mg (men)	24.17	22.45	1.07	209.02	214.22	0.97	119.6	116.69	1.02	Promote current dosing regimen
7-	25 mg (women)	28.04	23.19	1.2	275.78	188.08	1.46	90.64	132.92	0.68	
8-	86 mg	82.28	165.02	0.5	917.97	1827.5	0.5	93.68	47.05	1.99	
Pediatric after PO administration											
9-	0.556 mg/kg	44.32	44.14	1	339.38	329.31	1.03	1.73	1.79	0.96	Promote the current dosing regimen
10-	0.807 mg/kg	67.52	74.52	0.9	525.04	580.4	0.9	1.54	1.39	1.1	Promote the current dosing regimen
11-	1.25 mg/kg	94.95	81.16	1.16	823.91	467.25	1.76	1.51	2.67	0.56	Adjust doses to prevent toxicity
Adolescent after PO administration											
12-	0.930 mg/kg	74.16	89.1	0.83	649.49	839.07	0.77	1.39	1.07	1.29	Adjust doses to prevent su-therapeutic effect

C_max_: Maximal plasma concentration, AUC_0-∞_: Area under the concentration–time curve from 0-∞, CL: Clearance, Pre: Predicted, Obs: Observed, IV: Intravenous, PO: per-oral.

**Table 4:** AFE values in PO plasma/serum concentrations for all diphenhydramine adults (young and elderly) and pediatric PBPK models.

Average Fold Error (AFE)				
	Pharmacokinetic Variables			Clinical Interpretation
	C_max_	AUC_0-inf_	CL	
**Population**				
**Young adults**	0.83	0.97	1.05	Promote the current dosing regimen
**Elderly adults**	0.92	0.97	1.22	
**Pediatrics and adolescent**	0.97	1.11	0.97	

C_max_: Maximal plasma concentration, AUC_0-∞_: Area under the concentration–time curve from 0-∞, CL: Clearance, PO: Per-oral.

## Data Availability

Data are contained within the article.
